# Risk Assessment of BRONJ in Oncologic Patients Treated with Bisphosphonates: Follow-Up to 18 Months

**DOI:** 10.1155/2014/475859

**Published:** 2014-09-01

**Authors:** Scilla Sparabombe, Lucia Vitali, Alessandra Nori, Ricarda Sara Berlin, Marta Mazur, Giovanna Orsini, Angelo Putignano

**Affiliations:** ^1^Department of Clinical Sciences and Stomatology, Faculty of Medicine, Polytechnic University of Marche, Palace “Murri”, Floor No. 3, Via Tronto 10, 60126 Ancona, Italy; ^2^Special and Surgical Stomatology Department, “Ospedali Riuniti” Hospital of Ancona, Via Conca 2, 60126 Ancona, Italy; ^3^Stomatology and Maxillofacial Science Department, University of Rome “La Sapienza”, Italy

## Abstract

*Objectives.* Bisphosphonates related osteonecrosis of the jaw (BRONJ) is a pathological condition characterized by bone exposure or latent infection in patients treated with the drug. The aim of the study is to monitor the BRONJ level of risk health in patients with cancer, according to a preventive clinical protocol, which is firstly aimed at reducing risk factors such as the periodontal infections. *Materials and Methods.* 10 patients participated in the protocol and were evaluated at baseline and after 3 and 18 months of treatment with bisphosphonates, through full mouth plaque and bleeding scores (FMPS and FMBS), clinical attachment level (CAL) measurement, and the occurrence of osteonecrosis. *Results.* The mean plaque and bleeding were reduced and the CAL has not shown significant changes and in no cases was there manifestation of BRONJ. *Conclusion.* The protocol proved crucial for the maintenance of good oral health conditions by eliminating the risk of BRONJ during the observation period.

## 1. Introduction

Bisphosphonates are a group of drugs widely recommended and used for the treatment of moderate and severe hypercalcemia associated with cancer, for osteolytic lesions associated with metastases of breast cancer, prostate cancer, or multiple myeloma in combination with other chemotherapeutic agents. They are also used in the prevention and therapy of osteoporosis in postmenopausal women and also in the treatment of Paget's disease [1, 2].

These drugs are completely resistant to the hydrolytic cleavage, whereby this is the reason why they accumulate in the bone tissue and have a long half-life. Their rapid uptake in bone matrix allows an accumulation that goes from 30 to 70% of the administered intravenous dose or that absorbed after oral intake, while the remaining fraction is excreted unchanged into urine.

The accumulation of bisphosphonates in the bone, in particular in maxillary bones, is not reversible. Their toxic effect on osteoclasts depends on both the dose administered and the duration of therapy. The intravenous administration of high doses of aminobisphosphonates (N-BF), that is, the bisphosphonates of last generation containing nitrogen in the side chains, can cause the onset of necrosis of the jaw bone and/or of the mandibular bone [3, 4].

This pathology was identified with the acronym BRONJ (bisphosphonates related osteonecrosis of the jaw). It is a pathological condition described for the first time in 2007 [5] and in 2009 the AAOMS underlined that the presence of BRONJ is also discernible in the absence of bone exposure clinically detectable, by introducing a new stage of the disease: “stage 0” [6].

In 2012 Bedogni et al. [7] defined the BRONJ as an adverse reaction that is drug related, characterized by the destruction and necrosis of the jaw/maxillary bone in subjects treated with aminobisphosphonates, with no previous radiation treatment. On the basis of the recommendations published by the SICMF-SIPMO 2013 [8] “stage 0” was deleted by replacing in the other stages all cases without bone exposure.

The therapy of BRONJ is currently still a dilemma. In the literature unequivocally effective treatments have not been reported, and discontinuation of therapy with N-BF does not involve the healing of necrotic disease. The surgical approach, when indicated, is very aggressive and sometimes can cause a widening of the areas of bone exposure and amplify the symptoms.

The preventive approach is certainly the best way to avoid the onset of the disease. Particularly important in the prevention of BRONJ is the cofactors evaluation, that in the absence of bisphosphonates do not lead to the onset of the disease.

The knowing of BRONJ risk factors can be very helpful in planning a protocol. As suggested in the recommendations of the SICMF-SIPMO (Italian Society of Maxillofacial Surgery and Italian Society of Pathology and Oral Medicine), we do not yet have definitive data; certainly, taking the molecule N-BF is an high risk factor as well as the way of the administration: indeed, the risk increases in proportion to the dose administered intravenously.

Besides the cancer disease, which requires the recruitment of the molecule, seems to have a correlation with the increase of the risk. Another risk factor of BRONJ is the supporting therapy with antiangiogenics or with steroids. (Even if steroids are not able to produce osteonecrosis, they are undoubtedly cytotoxic and have an effect on the synthesis of collagen and then consequently wound healing. They also increase the toxicity factor of bisphosphonates.)

The local risk factors have also a relevant role; it is just in their knowledge that many of prevention strategies is based. On the basis of the data reported in the literature [8] the dentoalveolar surgery is the most important risk factor followed by the osteointegrated implants; the dentoperiodontal or peri-implant pathology is only the third one.

Among the local risk factors, periodontal diseases have a particular relevance. It is an inflammatory process induced by bacteria, causing an alveolar bone remodeling [9]; it strikes the adult population with a frequency of 90% [10]. In the case of recruitment of N-BF there is an inhibition of the resorption process in favour of a bone necrosis.

A recent study with rats [11] showed that, after administration of a dose of zoledronic acid, corresponding to the one accumulated in humans oncology therapies, and after inducing experimentally periodontal disease with sterile ligatures, the periodontal diseases, associated with the recruitment of zoledronic acid, are a necessary and sufficient condition to develop BRONJ.

The aim of this work has been to reduce the level of risk of BRONJ in patients with cancer and in therapy with aminobisphosphonates, before the recruitment, through a protocol targeted in a particular way at control of periodontal disease and the maintenance of oral health.

## 2. Materials and Methods 

The recruitment of patients occurred at the Surgical and Special Stomatology of the Neurological Medical Sciences Department, in the “Ospedali Riuniti” Hospital of Ancona, in the period from January 2012 to October 2012.

Since 2001 the structure adopts a protocol for the prevention of osteonecrosis (Table 1) [12, 13] in cooperation with the oncology, surgery, clinical medicine, and endocrinology division as synthetically reported:

- dental treatment before the therapy (phase I),
- dental treatment during the therapy, without bone disease (stage II), with bone necrosis (phase III),
- follow-up to 1 month–6 months.

This protocol is similar to the one proposed in the SICMF-SIPMO recommendations [8] updated to 2013 on the basis of the latest scientific evidences, in which it is possible to identify different paths depending on the type of patient and on the time in which it was intercepted. In the case of patients that have yet to start the recruitment of the drug it provides a path comparable to that described in Table 1 for phase I.

One of the main aspects, that comes out in all stages of this path, is the professional and the home oral hygiene care aimed at achieving and maintaining a state of health. The protocol has received the approval of the Marche Region Ethics Committee and is carried out in accordance with the ethical standards approved by the Declaration of Helsinki in 1964.

In 2012 43 oncology patients taken in care presented the following: 14% with lung cancer, 42% breast cancer, 23% multiple myeloma, 7% prostate cancer, and 7% bone metastases. The remaining 7% included oral carcinoma and cancer of the bladder, kidney, and colon. All patients read and signed, after careful and detailed verbal explanation, an informed consent included in the protocol of the department. In this standard format is also specified a consent to any use of the clinical data collected for scientific purposes.

All the patients were subjected to a dental visit (anamnesis; objective examination of intraoral and extraoral environment; assessment of removable prostheses; radiographic examinations) and were informed on the issues relating to the risk of the occurrence of BRONJ in relation to the level of oral health.

Carrying out a risk assessment was necessary to identify the BRONJ predisposing factors. For this purpose, each patient was subjected to questions about the diagnosis of cancer, the type and dosage of the drug administered, duration of therapy, and the presence of other drugs associated with the dental history and the oral habits (Table 2) [12].

For the present prospective study, patients were selected within 43 oncology patients, taken in care in 2012, and by considering the following inclusion criteria:

- people of both sexes,
- patients who must begin therapy with N-BF due to cancer or metastases,
- adults above the age of 30 years,
- complete or partial teeth,
- no manifestation of osteonecrosis,
- no radiotherapy of cervicofacial district.

Patients with the following were excluded:

- total edentulous,
- precarious conditions of general health (elderly patients very debilitated, patients undergoing recent surgical therapies, patients with nutritional deficiencies, patients with immune deficiency, and people who have cardiac and/or respiratory serious compromises),
- lack of collaboration,
- bisphosphonates therapy in act (phase II),
- clinical manifestation of BRONJ,
- no oncological diseases.

A decisive inclusion criterion of the study was the possibility to follow the patient throughout the period of observation at the hospital. In fact in most cases, once the phase I, the patient is entrusted to the territory for monitoring and maintenance.

After the visit (T0), all the patients were subjected to the following.

- Assessment of the visible plaque index [14] (in this text abbreviated with the acronym FMPS, i.e., Full Mouth Plaque Score, so called by Tonetti and his collaborators in 2002) and of the dichotomous bleeding index [14] (abbreviated form now on as FMBS), both drafted, as suggested by the international scientific literature, noting the positive sites and putting them in relationship with all of the sites examined.
- Assessment of the clinical attachment level (CAL); involvement of furcations; degree of dental mobility.
- Professional oral hygiene care.

All patients were instructed to perform correctly the oral hygiene at home, with particular attention to use nontraumatic tools and their association with mouthwashes that are alcohol-free.

There were also addressed the issues related to Hyposialism caused by the imminent pharmacology therapy: salivary substitutes, feeding and risk of caries, on the basis of a clinical protocol already existing [13].

Three sessions of maintenance and monitoring of oral health were made: (a) during therapy (T1); (b) at the end of the treatment with N-BF (T2) in which patients were subjected again to a session of professional oral hygiene care and to a reinforcement of education on oral hygiene care at home; (c) after 18 months from the start of therapy with N-BF (T3). The last phase included new probing and CAL, FMPS and FMBS reevaluation, and tissues and clinical signs control to exclude the occurrence of BRONJ. The data collected have been discussed and compared with the help of graphic representations. The CAL average was obtained through the use of software for the mathematical calculation.

For ethical reasons it was not possible to form a group of patients for the control.

## 3. Results

Out of 43 patients, 15 patients, belonging to phase 1 in 2012 and satisfying the criteria described above, were included. Due to a subsequent aggravation of the general state of health, 3 people have abandoned the study; 2 died during the observation period.

The 10 remaining patients, 7 females and 3 males, were aged between 38 and 78 years (50% over 70 years, one person less than 40 years, and 40% between 38 and 70 years) and all were to start therapy with N-BF for metastasis. The primary systemic pathology was breast cancer in 70% of the cases (7 women); two persons showed metastasis on colon and bladder.

Eight patients had to begin the periodic administration of intravenous zoledronic acid (Zometa), from 3 to 5 cycles every 28 days; 2 patients had to begin the ibandronic acid (Bondronat) by oral administration. All have completed the therapy with bisphosphonates. Six people have received the dose of 4 mg of zoledronate, pharmaceutically acceptable as a reconstituted and further diluted infusion (diluted with 100 mL of saline 0.9% w/v solution or glucose (5% w/v)), in at least 15 minutes for 3 administrations every 28 days; 1 patient received 4 doses every 28 days, and 1 person received 5 administrations of the drug with the same dosage and frequency.

Two patients have received an ibandronate daily dose of 2.5 mg per oral administration throughout the observation period (Table 3). In addition 2 patients were also subjected to chemotherapy, 2 patients were subjected to administration of corticosteroids, and 4 patients have carried out radiotherapy, at the end.

The main preexisting dental pathology proved to be the generalized chronic periodontitis and, in fact, it is present in 70% of patients. In one case apical granulomas were detected and a couple of patients also showed radicular residues.

The initial level of risk of the subjects is described in Table 4: all patients were considered at high risk of developing BRONJ. This evaluation was carried out on the basis of the high dosage of drug taken during the period of observation and on the conditions of oral health detected during the first visit.

In the first visit (T0), 4 patients out of 10 had a level of oral hygiene, expressed with the index FMPS, higher than 90%, 5 showed percentages ranging between 40 and 70%, and only one patient had a visible plaque index of 24%; the average index was 73%.

Nine people needed a tooth extraction and all were subjected to one or more sessions of professional oral hygiene before starting therapy with N-BF.

**Figure 1:**
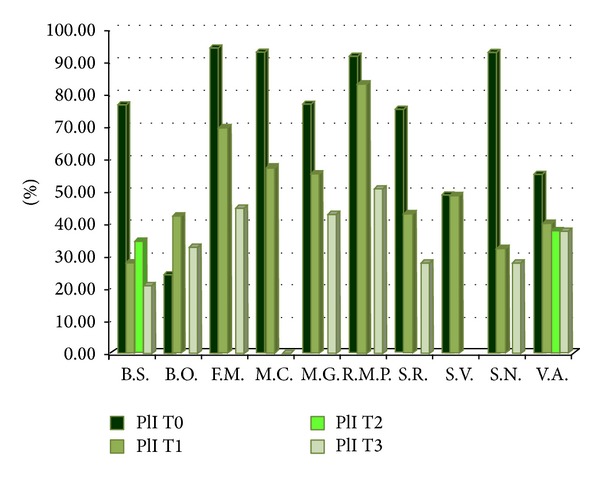
FMPS detected before the start of therapy (T0) and 3 months (T1), 6 months (T2), and 18 months (T3) after. The patient S.V. died before the follow-up at 18 months.

In the second control (T1), 3 months after the start of therapy, the average of the FMPS has suffered a considerable reduction coming to 50%. Only two patients have participated after 6 months in a further follow-up (T2) expressing an average percentage of 36% FMPS. The last control, performed 18 months from the beginning of therapy (T3), has been detected on 9 patients because of a supervening death. The plaque index average was 29%.  Figure 1 shows the evolution of the 10 patients in the time of observation. The gingival inflammation, expressed through the FMBS, shows a sample less homogeneous with respect to the oral hygiene level.

**Figure 2:**
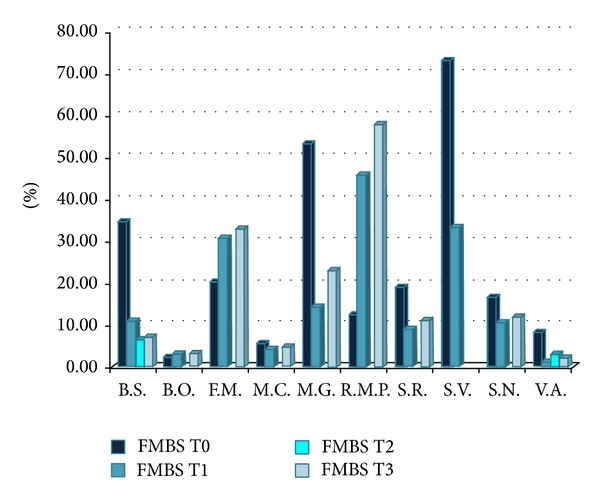
FMBS detected before the start of therapy (T0) and 3 months (T1), 6 months (T2), and 18 months (T3) after. The patient S.V. died before the follow-up at 18 months.

At the first visit two patients had a FMBS greater than 50%; six out of 10 people had a percentage lower than 20% and the average is 24%. Subsequent checks showed, at 3 months, a FMBS average of 16%; at 6 months the two patients monitored had an average less than 5% and at 18 months the average of 9 people was 15%.  Figure 2 shows the overall trend of FMBS in the sample examined.

**Figure 3:**
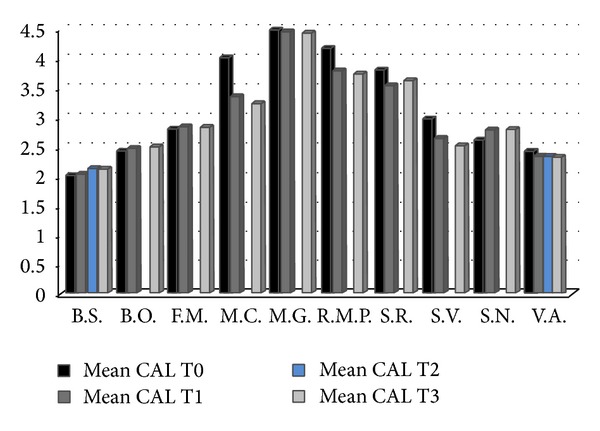
CAL average before and during therapy and type of drug administered.

The periodontal exam has highlighted the presence of a periodontal impairment with loss of clinical attachment (CAL) in all the patients: range of 2 to 4.5 mm, average of 3.15 mm. At the first follow-up the CAL average dropped to 2.9 mm and in the last control (18 months) it was 2.8 mm (Figure 3).

The last visit (follow-up at 18 months) was made through clinical examination and radiographic examination and revealed the total absence of signs of osteonecrosis in all patients.

## 4. Discussion

Osteonecrosis today affects about 20,000 people a year [15]. The BRONJ are complications that affect 2.8% of patients who receive N-BF for bone metastases of breast cancer [16]. The sample selected for this study, although small, is therefore representative of the most risk of osteonecrosis.

On the basis of the first reports, the literature identified BRONJ only in relation to oral surgical access to the maxillary bones (extractions) [16, 17]. Today it tends to emphasize the importance of the presence of periodontal disease, latent or not fully treated, such as infection triggers of BRONJ [18–20].

In all cases of BRONJ treated by Marx et al. [4], the 25% of the lesions were found to be arising spontaneously, while 75% were engendered by some type of dental invasive procedure. More precisely, Marx indicates that, in 152 patients with BRONJ, more than a third, a trigging factor was due to tooth extractions. Of these, about half, was caused by periodontal disease, of which 26% was represented by untreated parodontitis, and in 25% of the cases, it seemed to be a manifestation of the osteonecrosis which the author calls “spontaneous.” The latter confirmed the hypothesis that there is no doubt that the subclinical osteonecrosis also exists [21] even if there is no bone exposure. This justifies the assertion of many authors that the prevalence of BRONJ has not yet been established and its pathogenesis is not entirely clarified [18].

In the present study, the first visit revealed in all the patients the presence of oral preexisting diseases and the most popular is periodontitis [22]. The presence of this disease, manifest or latent, associated with bacterial plaque and calculus and inadequate oral hygiene; it can certainly be regarded as a serious risk factor for the onset of BRONJ [23].

The risk of developing BRONJ for these patients, in phase 1 of the protocol (T0), was judged to be very high especially in relation to the high dose of the drug taken during the period of observation and to the conditions of oral health detected during the first visit.

Optimizing oral health should therefore be the primary objective; teeth that are not treated or teeth with a poor prognosis must be extracted by delaying the start of therapy with N-BF at least 4–6 weeks to ensure complete healing of the tissues. Patients should be instructed on the importance of good hygiene at home and motivated to undergo regular checks of monitoring and maintenance.

After the first preventive intervention (T0) Figure 1 shows a general progressive reduction of the plaque index.

It is necessary to emphasize that the sample is composed of elderly people. It was possible to confirm a general improvement in the level of oral hygiene even if the educational intervention in these patients is very difficult, not only because of the age but also because often their interest is focused on pain, on the therapies that must be undergone, on emotional factor that comprises the concern for the sick, and on the outcome of care.

Most patients, during the administration of the drug, have suffered from fever, severe joint pain, general malaise, and gastrointestinal problems with consequent general debilitation. Such symptoms are immediately manifested after administration and are attenuated during the following days. In this context to speak about toothbrush and proxabrush may seem irrelevant. A correct psychological approach and respect of each patient's limits should be necessary.

At T1 the FMPS and FMBS percentages decreased, except some exceptions. In two cases the bleeding index, in the second control, resulted higher than those on the first check; it is not to exclude an effect of the drug on gingival tissue.

As regards the CAL, in the subsequent controls, differences are not significant (Figure 3) but they show the slight packaging of tissues following the periodontal therapy. It could indicate a constant maintenance of the level of periodontal health and the absence of periodontal pockets or latent osteonecrosis.

In three patients showing a greater reduction of CAL from T0 to T1, it is reasonable to assume a reduction in the depth following the professional oral hygiene. There seems to be no difference between patients who were taking N-BF intravenous and by oral administration.

The data collected show that patients observed in T0 showed a high level of risk disease; this risk was significantly reduced once included in the protocol of prevention of BRONJ. These considerations justify the result reached after 18 months, when the follow-up evaluation shows patients with good oral health and total absence of BRONJ.

## 5. Conclusions

BRONJ represents an unwanted complication of N-BF and its prevention begins with the close cooperation of the following specialists: oncologist, rheumatologist, maxillofacial surgeon, dentist, and dental hygienist.

In the light of the data and clinical observations reported in the present study, it is conceivable that the protocol applied and described above has been important in cancelling the incidence of the disease in the group of patients examined, that is, group considered at high risk of BRONJ.

Today the occurrence of BRONJ is calculated on the bases of retrospective studies putting it in a range from 8% to 11% [24], but these percentages are increasing. The low number of observed patients and the lack of a group of control (excluded from protocol for ethical reasons) call for further depths even if this work suggests the big importance of the preventive approach.

## Figures and Tables

**Table 1:** Clinical protocol for the integrated care for oncology patient implemented from 2001 in Surgical and Special Stomatology Division—Ospedali Riuniti Hospital of Ancona [12, 13].

Protocol for the integrated care for oncology patient			
Diagnostic section	Anamnesis		
	Clinical examination		
	Oral radiographic		
	Indices of oral health		
	Periodontal status		
	Photographic documentation		

Therapeutic section	Treatment before starting N-BF therapy step 1	Treatment during N-BF therapy steps 2 and 3	Follow-up
	(i) First visit (ii) RX exams (iii) Tooth extractions, endodontics, and restorative (iv) Professional oral hygiene and education about the oral hygiene at home (v) Prophylaxis of caries (vi) Instructions about complications and awareness of the problem	(i) Adaptation of symptomatic and preventive therapy—follow-up oral hygiene to 15gg—1 month (ii) Follow-up tissues and clinical signs at 3-4 months	Oral health evaluation and professional hygiene symptomatic therapy of the secondary effects—prophylaxis of caries 1–3 months—follow-up to 1–6 months

**Table 2:** Information to identify the risk factors for the development of BRONJ [12].

Risk factors	Description
Diagnosis of malignant neoplasia	(i) Type of cancer (ii) Presence of metastases and localization previous therapy (surgery, radiotherapy)

Drug administered	(i) Type (ii) Total dosage (iii) Recruitment (iv) Timing of therapy

Other drugs	(i) Corticosteroids (ii) Antiangiogenic

Oral history	(i) Traumas (ii) Surgical procedures (iii) Dental and gum infections (iv) Diagnosis of periodontal disease (v) Implantology (vi) Prosthesis

Oral hygiene	(i) Daily home care (ii) Annual frequency professional care (iii) Motivation and information level

**Table 3:** Type of drug, administrations, and doses linked to systemic pathology.

Pathology	Drug	Dosage
Lung cancer + bone metastases	Zoledronate	4 MG × 3 administrations every 28 days
Prostate + bladder cancer + bone and lymph node metastases	Zoledronate	4 MG × 3 administrations every 28 days
Breast cancer + bone metastases	Zoledronate	4 MG × 3 administrations every 28 days
Breast cancer + bone and lymph node metastases	Zoledronate	4 MG × 3 administrations every 28 days
Lung cancer + bone metastases	Zoledronate	4 MG × 5 administrations every 28 days
Breast and colon cancer + bone metastases	Ibandronate	2,5 mg by os/day
Breast cancer + bone and lung metastases	Zoledronate	4 MG × 3 administrations every 28 days
Breast cancer + bone metastases	Ibandronate	2,5 mg by os/day
Breast cancer + bone metastases	Zoledronate	4 MG × 3 administrations every 28 days
Breast cancer + bone, pulmonary and hepatic metastases	Zoledronate	4 MG × 3 administrations every 28 days

**Table 4:** Risk evaluation to T0 (bold = high risk, italic = low risk, and bold italic = not definable risk).

		P. 1	P.2	P.3	P.4	P.5	P.6	P.7	P.8	P.9	P.10	
Aminobisphosphonates molecule	**Zoledronate**	X	X	X	X	X	X	X	X			Not changeable risk factors
	*Ibandronate *									X	X	
Other medicines/therapies	**Subsequent chemotherapy**			X		X						
	**Subsequent radiotherapy**				X			X	X		X	
	**Concomitant corticosteroids administration **	X	X									
Administration	**Intravenous**	X	X	X	X	X	X	X	X			
	***Oral***									X	X	
Systemic factors	**Presence of cancer**	X	X	X	X	X	X	X	X	X	X	

Local risk factors	**Periodontal pathology**	X	X	X	X	X		X			X	Modifiable risk factors
	**Dental pathology **	X	X		X	X	X	X	X	X	X	
